# Physical function and severe side effects matter most to patients with RA (< 5 years): a discrete choice experiment assessing preferences for personalized RA treatment

**DOI:** 10.1186/s41927-023-00341-y

**Published:** 2023-07-03

**Authors:** Karin Schölin Bywall, Bente Appel Esbensen, Marie Heidenvall, Inger Erlandsson, Marta Lason, Mats Hansson, Jennifer Viberg Johansson

**Affiliations:** 1 School of Health, Care and Social Welfare, Division of Health and Welfare Technology, Mälardalen University, Västerås, Sweden; 2 Department of Public Health and Caring Sciences, Centre for Research Ethics & Bioethics, Uppsala University, Uppsala, Sweden; 3 Copenhagen Center for Arthritis Research (COPECARE), Center for Rheumatology and Spine Diseases, Centre of Head and Orthopaedics, Rigshospitalet, Copenhagen, Denmark; 4 Department of Clinical Medicine, Faculty of Health and Medical Sciences, University of Copenhagen, Copenhagen, Denmark; 5 Rheumatism Association, Stockholm, Sweden; 6 Elsa Science, Stockholm, Sweden; 7 Institute for Future Studies, Stockholm, Sweden

**Keywords:** Precision medicine, Rheumatoid arthritis, Individualised treatment, Shared decision-making

## Abstract

**Aim:**

Early assessment of patient preferences has the potential to support shared decisions in personalized precision medicine for patients with rheumatoid arthritis (RA). The aim of this study was to assess treatment preferences of patients with RA (< 5 years) with previous experience of inadequate response to first-line monotherapy.

**Method:**

Patients were recruited (March–June 2021) via four clinics in Sweden. Potential respondents (*N* = 933) received an invitation to answer a digital survey. The survey included an introductory part, a discrete choice experiment (DCE) and demographic questions. Each respondent answered 11 hypothetical choice questions as part of the DCE. Patient preferences and preference heterogeneity were estimated using random parameter logit models and latent class analysis models.

**Results:**

Patients (*n* = 182) assessed the most important treatment attributes out of physical functional capacity, psychosocial functional capacity, frequency of mild side effects and likelihood of severe side effects. In general, patients preferred a greater increase in functional capacity and decreased side effects. However, a substantial preference heterogeneity was identified with two underlying preference patterns. The most important attribute in the first pattern was the ‘likelihood of getting a severe side effect’. Physical functional capacity was the most important attribute in the second pattern.

**Conclusion:**

Respondents focused their decision-making mainly on increasing their physical functional capacity or decreasing the likelihood of getting a severe side effect. These results are highly relevant from a clinical perspective to strengthen communication in shared decision making by assessing patients’ individual preferences for benefits and risks in treatment discussions.

**Supplementary Information:**

The online version contains supplementary material available at 10.1186/s41927-023-00341-y.

## Key points

▪ This study reveals that patients with RA (< 5 years) and inadequate response to first-line monotherapy have differing treatment preferences. They focus mainly on increasing functional capacity or on the likelihood of getting a severe side effect.

▪ Therefore, communication in shared decision-making needs to account for heterogeneity in patient preferences in order to increase patient-centeredness in personalized precision medicine.

▪ Future research needs to develop tools to strengthen patients in making shared decisions with health care professionals in order to improve personalized precision medicine.

**Supplementary Information:**

The online version contains supplementary material available at 10.1186/s41927-023-00341-y.

## Introduction

Precision medicine aims to predict how patients will respond to a specific therapy. These prediction algorithms are based on individual characteristics such as genetic factors, age, health status, environmental exposure, concurrent therapies, etc. [1]. The main goal of precision medicine in rheumatoid arthritis (RA) is to tailor the treatment strategy based on individual patient characteristics and to help physicians and patients in setting treatment goals [2, 3]. This strategy requires tight disease control with frequent assessments of the patient and adjusting treatment until the goal is reached and sustained [4]. Shared decision-making is important in all aspects of RA care, to increase compliance and improve treatment outcomes [5]. Ideally, the decision to alter treatment should be aligned with both physicians’ clinical recommendations and patients’ personal preferences [6].

Shared decision-making requires patients and physicians to evaluate potential treatment alternatives to align decisions with both clinical and individual treatment goals and preferences [7]. RA is characterised by symptoms of pain, stiffness and fatigue [8]. The chronic and progressive nature of this autoimmune joint disease has a major and long-lasting effect on quality of life. Patients will need life-long pharmacological treatment associated with potential serious side effects [9]. The unpredictable course of the disease may also require patients to adjust to an altered functional capacity, due to limitations in almost all areas of daily life, including work and physical and social activities. In addition to its effect on everyday life, the disease process itself may further influence psychosocial functional capacity due to a direct link between inflammatory processes and depressive symptoms [10].

Assessing patients’ individual preferences is a central part of making shared treatment decisions in personalized precision medicine for patients with early RA and inadequate response to first-line monotherapy to achieve therapeutic success [11]. Identifying how patients with RA trade improvements in functional capacity against potential side effects may be an innovation for shared decision-making that has the potential to support physicians in addressing patient preferences during clinic visits [5]. Therefore, quantitative assessments of patient-relevant benefits and risks have the potential to align precision medicine with patient preferences [12]. The aim of this study was to assess the treatment preferences of patients with RA and inadequate response to first-line monotherapy.

## Methods

A discrete choice experiment (DCE) was used to quantify patient preferences by revealing the relative importance of treatment attributes and underlying patterns of preferences [13]. Patients with RA (< 5 years) received an invitation by post to respond to a digital patient preference survey assessing treatment preferences.

### Respondents and recruitment

Patients with RA were recruited via four rheumatology clinics in Sweden. Potential respondents were eligible for the study if they had an established RA diagnosis (< 5 years), were between 18 and 80 years of age, had inadequate response to first-line monotherapy with Methotrexate by lack of sufficient treatment effect or unbearable side effects, and were able to read and understand the questions on their own. The rationale for this inclusion was the aim to assess patients’ preferences by giving the respondents hypothetical choice scenarios of needing to change treatment due to inadequate response to first line-treatment, by using the discrete choice experiment method (DCE). We included respondents that had real experience within the last 5 years to be part of the real scenario, i.e., they had a previous inadequate response to Methotrexate, and they had to change treatment strategy.

As a first step, eligible patients were identified via the Swedish Rheumatology Quality Register. Second, an invitation to participate in the digital survey was sent out by post between March and June 2021. The invitation included information about the study, a link to the digital survey and a unique password. The invitation letter went out to around 933 potential respondents. This survey was approved by the regional ethics review board in Uppsala, Sweden (Reg no. 2020/00556). Data collection and recording, storage, and dissemination were governed by the General Data Protection Regulation and Uppsala University’s data protection and security policies. All respondents gave informed consent.

### Discrete choice experiment

The main part of this survey was the DCE, used to assess treatment preferences of patients with early RA (< 5 years) and previous inadequate response to first-line monotherapy in Sweden [13]. This method is grounded in random utility theory, which suggests that individuals make rational choices to maximise utility for themselves. A value is assigned, defined as the sum of an individual's utilities of predefined attributes. This value is estimated as a function of underlying features [14]. Respondents in this DCE had to make repeated choices between two alternatives (i.e., choice questions) that were characterised by different attribute levels.

### DCE development

Attributes were identified and selected in co-creation with patients and health care professionals, based on methodological recommendations [15]. Attributes were initially identified through a scoping literature review. The review encompassed articles relevant for assessing preferences among patients with RA, to get an insight into commonly used attributes. In total, 373 article abstracts were screened for potential attributes. Of these, 23 were eligible for inclusion in attribute identification after full review [6, 16–37].

Patient-relevant attributes were further filtered and validated with patient research partners (MH, IE), health care professionals (BAE and external members of the team) and preference researchers (KB, JVJ). In April 2020, nine potential attributes were ranked by patients with RA (*N* = 185) using a mobile application (www.elsa.science.se), to further guide the selection of attributes. The attributes ranked were: route of administration, reduced inflammation, improved functional capacity, reduced pain and fatigue, risk of mild side effects, risk of side effects changing appearance, risk of psychological side effects, risk of severe side effects and risk of long-term damage. Semi-structured interviews were conducted with patients with RA (*N* = 10) to further select and frame the most important attributes and levels to be assessed in the DCE. The interviews revealed two dimensions (physical and psychosocial) of the highest ranked attribute ‘functional capacity’. Through the ranking exercise and interviews, mild and severe side effects were also identified as being among the most important attributes that patients considered in treatment decisions [38]. Refinement of the potential attributes and levels was carried out through discussions within the research team (i.e., including patients, health care professionals and researchers) and with four external rheumatologists. A total of four attributes with 3–4 levels each were selected for inclusion in the DCE (Table 1). Respondents were instructed to imagine themselves in a position of not having a well-functioning treatment and to select the alternative (treatment A or B) that best aligned with their individual preferences.

**Table 1:** Attributes and levels in choice questions

Attribute	Level 1 (ref)	Level 2	Level 3	Level 4
**Physical functional capacity**: My ability to perform daily physical chores and activities (such as work, studies and household, family and leisure activities). Imagine a scale where the starting point is when your treatment is not working. The distance to full physical functional capacity is presented as percentages with the following levels:	Improved by 25%	Improved by 50%	Improved by 75%	Full functional capacity 100%
**Psychosocial functional capacity**: How I feel about life and my ability to manage daily psychosocial activities. Imagine a scale where the starting point is when you feel mentally bad about yourself. The distance to full psychosocial functional capacity is presented as percentages with the following levels:	Improved by 25%	Improved by 50%	Improved by 75%	Full functional capacity 100%
**Frequency of mild side effects**: Mild side effects are often temporary and go away after a couple of days. Some examples of mild side effects are headache and nausea. People can experience side effects in different ways. The levels are described as:	Low, a few times during a 3-month	Medium, a few times during a month	High, a few times during a week period	
**Likelihood of severe side effects**: The likelihood of getting a severe side effect, such as a serious infection or an allergic reaction can differ between therapies. The levels are described as:	Rare, 1 out of 1,000 can get the side effect	Common, 1 out of 100 can get the side effect	Very common, 1 out of 10 can get the side effect	

### Experimental design

**Fig. 1:**
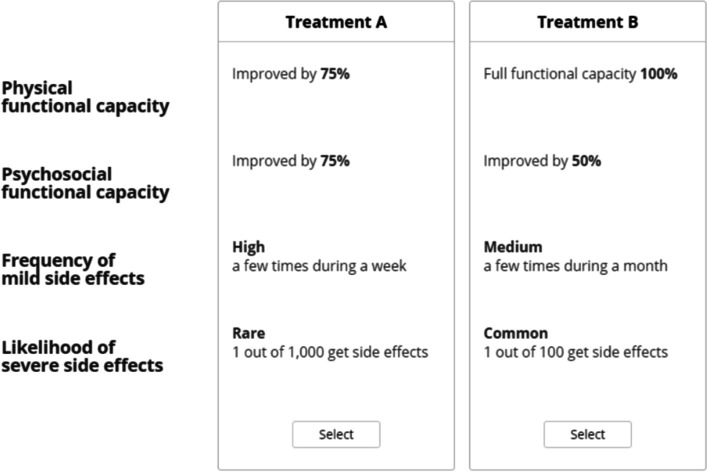
Example of a choice question

An experimental design for the DCE was constructed in NGene 1.0 (ChoiceMetrics, 2011). Each respondent was asked to answer 11 hypothetical choice questions (Fig. 1). Each choice question included two pairwise comparisons that were characterised by different attribute levels. Three patients with RA were interviewed to improve and validate survey comprehension and relevance before the pilot. Then, an invitation was sent by email to patients with RA, with a link to the survey. The interviewees answered the survey during a digital meeting with the first author KSB, to improve comprehension and framing of the attributes and levels. The respondents were encouraged to ‘think aloud’ while answering the survey. Based on the data retrieved in the pilot test (*n* = 24), a multinomial logit (MNL) model was fitted. Beta estimates were used to assign priors for the final experimental *d*-efficient (Bayesian) design [15]. The final design included 33 choice questions divided into three blocks and was implemented in a DCE using Lighthouse Studios v 9.8.1.

### Statistical analysis

Demographic questions were analysed using descriptive analyses and presented as frequencies and percentages. Patient preferences were estimated using multivariate methods: a MNL model, a random parameter logit (RPL) model and latent class models [39]. SPSS and Nlogit were used for statistical analyses. Statistical tests were conducted at the 5% significance level and corresponding 95% confidence intervals were presented. All the attributes were dummy-coded (i.e., normalised to 0). Likelihood ratio tests and the Akaike information criterion were used to check the accuracy of the models, to determine the most appropriate model and to test for parameters that might improve the model fit [40].

A MNL model was created to account for the multilevel structure of the data. The MNL model revealed that all the attribute estimates significantly contributed to the decision-making process of respondents. The RPL model allowed further analysis of heterogeneities within the individual attributes and levels. The RPL model took unobserved heterogeneities between the survey participants into account. Because the RPL model adopted ‘random parameters’, it allowed an analysis of heterogeneities within the individual attributes and levels within the model. The extent of heterogeneous preferences within the sample was inferred from the standard deviations (SDs). The RPL model revealed significant SDs for most levels, as a result of heterogenous preferences within the sample. Therefore, exploratory analysis was generated by means of latent class models [39]. The latent class analysis (LCA) divided the population into underlying ‘latent’ preference classes with a probability of belonging to a certain class [41]. Several demographic and disease-related variables were tested for their potential impact on class membership in the LCA: age, gender, occupation, education, numeracy, health literacy, RA duration, treatment, side effects, health status, pain, worry and compliance (see Supplementary file).

The relative importance of each attributes was calculated as the difference between the highest and lowest estimates of the level for that attribute. The largest difference value was given a 1, representing the most important attribute. All other values were divided by the largest difference value (i.e., 1), resulting in a relative distance between all attributes.

## Results

In total, 182 patients with RA (< 5 years) and previous inadequate response to first-line monotherapy were included in the analysis out of the 933 that received the invitation (17%). Respondents were included if they answered to the whole questionnaire and excluded if they finished the survey under 5 min. Most of the respondents were female (74%), highly educated (59%) and diagnosed with RA 2–4 years before the study (Table 2). The age span ranged from 18 to 80 years and the majority of the respondents reported the time to drug effect as < 12 months. Most of the respondents (88%) had tried at least one first-line synthetic DMARD. Respondents had experienced mild side effects (68%), side effects affecting appearance (43%), psychological side effects (39%) and severe side effects (11%).

**Table 2:** Demographic and disease characteristics of patient population

	*N* = 182 (%)
**Gender**	
Female	129 (74)
Male	45 (26)
Other	1 (1)
**Age**	
18–34	14 (8)
35–44	52 (30)
55–64	51 (29)
65–80	57 (33)
**Educational level**	
No formal schooling or Elementary school	39 (22)
High school or Vocational training	32 (18)
University	104 (59)
**Current health status**	
Very good	27 (15)
Good	72 (41)
Okay	64 (37)
Poor	12 (7)
**RA duration**	
0–2 years	28 (16)
2–3 years	101 (58)
3–4 years	36 (21)
> 5 years	10 (6)
**The length of time for current medicine to start working**	
0–3 months	79 (48)
3–12 months	54 (33)
1–2 years	12 (7)
2–5 years	17 (10)
Still no effect	4 (2)
**Ever prescribed DMARDs**	
Synthetic DMARDs	159 (88)
Biologic DMARDs	63 (35)
JAK inhibitors	15 (8)
**Ever experienced side effects**	
Mild side effects	115 (64)
Side effects affecting appearance	78 (43)
Psychological side effects	71 (39)
Severe side effects	19 (11)
**How pleased are you with your treatment?**	
Very pleased	64 (35)
Somewhat pleased	94 (54)
Somewhat displeased	11 (6)
Very displeased	8 (5)
**How often do you take your treatment based on prescriptions from rheumatologists?**	
Always	152 (87)
Often	19 (11)
Sometimes	1 (1)
Never	3 (2)
**Level of physical function**	
I can walk without difficulties	129 (74)
I can walk with some difficulties	44 (25)
I am on bed rest	1 (1)
**Handling personal hygiene**	
I don’t need any help with my daily hygiene	162 (93)
I have some difficulties in washing or dressing myself	12 (7)
I can’t wash or dress myself	0
**Ability to perform daily tasks and activities**	
I can manage my daily activities	138 (79)
I have some problems in managing my daily activities	34 (20)
I can’t manage my daily activities	2 (1)
**Experience of pain**	
I have no pain	31 (18)
I have some pain	141 (81)
I have a lot of pain	2 (1)
**Experience of worry**	
I am not worried	105 (60)
I am worried to some extent	66 (38)
I am a worried to a large extent	3 (2)

### Patient preferences in RA treatment

Respondents in general preferred increased functional capacity and decreased side effects (i.e., the sign of the beta indicated that functional capacity had a positive impact on patient choices and side effects had a negative impact). The most important attribute (based on the relative importance (RI) scored from the RPL model, see Table 3) was ‘increase in physical functional capacity’ (RI = 1), followed by ‘likelihood of severe side effects’ (RI = 0.78), ‘increase in psychosocial functional capacity’ (RI = 0.35), and ‘frequency of mild side effects’ (RI = 0.33) (Table 3).

**Table 3:** Random parameter logit model

Attribute levels	Estimate (SE)	*P* value	CI	SD	*P* value (SD)	SE (SD)	CI (SD)	RI
**Increase in physical functional capacity**								1
25% improvement (ref^a^)								
50% improvement	2.32 (0.43)	< 0.01	1.47 – 3.16	0.19	N/A^b^	0.33	-0.45 – 0.84	
75% improvement	3.49 (0.45)	< 0.01	2.60 – 4.36	0.16	N/A	0.28	-0.38 – 0.70	
100% full functional capacity	4.59 (0.53)	< 0.01	3.55 – 5.62	0.97	< 0.01	0.27	0.44 – 1.48	
**Increase in psychosocial functional capacity**								
25% improvement (ref)								0.35
50% improvement	0.83 (0.25)	< 0.01	0.34 – 1.32	0.02	N/A	0.34	-0.65 – 0.68	
75% improvement	1.05 (0.26)	< 0.01	0.53 – 1.57	0.31	N/A	0.22	-0.12 – 0.73	
100% full functional capacity	1.62 (0.29)	< 0.01	1.055 – 2.18	0.65	< 0.01	0.21	0.22 – 1.06	
**Frequency of mild side effects**								0.33
Low (ref)								
Medium	-0.41 (0.15)	< 0.01	-0.71 – (-0.11)	0.62	< 0.01	0.21	0.20 – 1.00	
High	-1.52 (0.21)	< 0.01	-1.92 – (-1.11)	1.30	< 0.01	0.17	0.96–1.63	
**Likelihood of severe side effects**								0.78
Rare: 1 in 1,000 (ref)								
Common: 1 in 100	-0.94 (0.22)	< 0.01	-1.37 – (-0.49)	0.52	< 0.05	0.22	0.09 – 0.94	
Very common: 1 in 10	-3.58 (0.39)	< 0.01	-4.35 – (-2.80)	2.64	< 0.01	0.31	2.04 – 3.22	

^a^Reference category

^b^N/A: not applicable

### Preference heterogeneity among RA patients

A substantial preference heterogeneity, with two latent preference patterns, was identified using the LCA model (Table 4). The preference pattern in the first group (43%) was characterised by a very high rating of the likelihood of getting a severe side effect, followed by increase in physical functional capacity, frequency of getting mild side effects, and increase in psychosocial functional capacity. In the other pattern (57%), increase in physical functional capacity was the most important attribute, followed by increase in psychosocial functional capacity, frequency of mild side effects and likelihood of severe side effects. None of the disease-related variables that were tested had an impact on class membership in the LCA (see supplementary file for covariate estimates).

**Table 4:** Latent class analysis

Attribute levels	Class 1 estimate (SE)	*P* value	CI	RI	Class 2 estimate (SE)	*P* value	CI	RI
**Increase in physical functional capacity**				0.50				1
25% improvement (ref^a^)								
50% improvement	1.0 (0.68)	N/A^b^	-0.33 – 2.32		1.98 (0.48)	< 0.01	1.04 – 2.92	
75% improvement	2.08 (0.70)	< 0.01	0.71 – 4.45		2.95 (0.52)	< 0.01	1.93 – 3.97	
100% improvement	2.36 (0.81)	< 0.01	0.76 – 3.94		3.81 (0.60)	< 0.01	2.62 – 4.98	
**Increase in psychosocial functional capacity**				0.10				0.36
25% improvement (ref)								
50% improvement	0.11 (0.42)	N/A	-0.71 – 0.94		0.68 (0.25)	< 0.01	0.19 – 1.16	
75% improvement	-0.12 (0.41)	N/A	-0.92 – 0.67		1.12 (0.29)	< 0.01	0.56 – 1.68	
100% improvement	0.45 (0.44)	N/A	-0.41 – 1.30		1.36 (0.27)	< 0.01	0.82 – 1.89	
**Frequency of mild side effects**				0.13				0.34
Low (ref)								
Medium	-0.13 (0.28	N/A	-0.68 – 0.40		-0.46 (0.16)	< 0.01	-0–77 – (-0.14)	
High	-0.61 (0.31)	< 0.05	-1.21 – (-0.00)		-1.29 (0.21)	< 0.01	-1.69 – (-0.88)	
**Likelihood of severe side effects**				1				0.31
Rare: 1 in 1,000 (ref)								
Common: 1 in 100	-0.94 (0.42)	< 0.05	-1.76 – (-0.11)		-0.38 (0.23)	N/A	-0.83 – 0.07	
Very common: 1 in 10	-4.69 (1.09)	< 0.01	-6.82 – (-2.56)		-1.17 (0.31)	< 0.01	-1.77 – (-0.07)	
Average class probability	43				57			

^a^Reference category

^b^N/A: not applicable

### Relative importance and different aspects of RA treatment

**Fig. 2:**
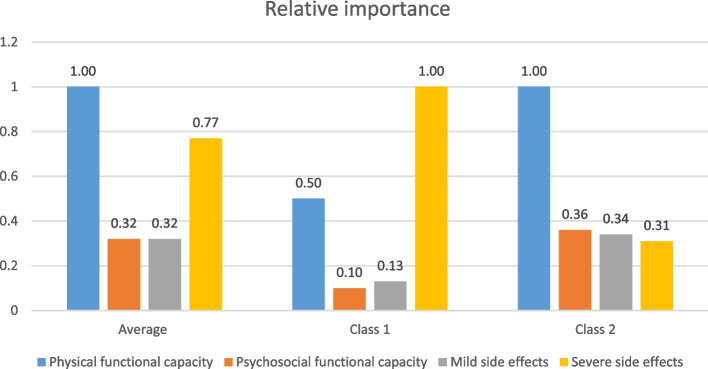
Relative importance of treatment attributes based on latent class analysis

On average (i.e., relative importance adjusted for class probability), the most important attribute was increase in physical functional capacity, followed by likelihood of getting a severe side effect (Fig. 2). Preference heterogeneity in the two latent preference patterns was estimated by calculating the RI scores. The likelihood of getting a severe side effect (infection or allergic reaction) was the most important attribute in class 1 (0.34%) respondents. The following order of attributes was normalised to the most important (i.e., 1): increase in physical functional capacity (0.50), frequency of mild side effects (0.13) and increase in psychosocial functional capacity (0.10). In class 2 respondents, the most important attribute was increase in physical functional capacity (1), followed by increase in psychosocial functional capacity (0.36), frequency of mild side effects (0.34) and likelihood of severe side effects (0.31).

## Discussion

The aim of this study was to assess the treatment preferences of patients with RA (< 5 years) and previous inadequate response to first-line monotherapy. The results of the study suggested that patients with RA (< 5 years) had differing treatment preferences.

The most important treatment attribute in latent class 1 was the ‘likelihood of getting a severe side effect’ when weighted against all other treatment attributes in the DCE. In latent class 2, the ‘increase in psychosocial functional capacity’ was the most important treatment attribute. These results are highly relevant from a clinical perspective as patient preferences are recognised as essential components of shared decision-making in international treatment guidelines for RA [9].

Although general treatment guidelines provide a framework to inform treatment decisions and underline the importance of shared decision-making between patients and health care professionals, communication often fails due to differing standpoints [42]. Such preference heterogeneity (Fig. 2) underlines the importance of physicians recognising individuals’ own preferences (not the average preferences of the patient population) and accounting for that in communication about treatment goals and strategies.

Making shared treatment decisions is important because of the potential to improve clinical outcomes, overcome patients’ resistance to altering treatment and improve treatment compliance and satisfaction [5]. A shared understanding that involves tight assessments of disease activity, treatment strategies and individual preferences may improve communication between patients and clinicians and might lead to higher decision quality. Results from our study could support communication in shared decision making to account for heterogeneity in individual patients preferences’ by addressing the multidimensional span of treatment goals (i.e., physical and psychosocial) affecting everyday life, to achieve a sense of ‘a normal life’ despite the presence of chronic illness [43].

Multidimensional intervention programmes are needed to increase shared decision-making in treatment decisions, which is particularly important given the chronic nature of RA, where patients usually administer their own treatment [44]. Such interventions could focus on self-efficacy and patients’ belief in their own ability to administer treatment and be involved in treatment decisions. High self-efficacy is associated with good patient-doctor communication and patients feeling more capable of taking medications appropriately [45]. Our study has revealed significant aspects (functional capacity and side effects) to focus on when designing such intervention programmes.

Multidimensional intervention programmes also need to educate patients about treatment alternatives and promote reflection on individual treatment preferences for shared decision-making to work in clinical practice. As seen in recent shared decision interventions, digital decision aids, such as mobile health applications, may be used to inform patients about potential treatment alternatives and support patients in reflecting on their individual treatment preference and goals as a part of self-management [44]. Digital decision aids have supported patients with RA in making informed decisions when they have little or no experience with the choice situation [23]. The results from our study highlighting patients’ different treatment preferences should be considered when designing shared decision interventions, to account for individual treatment goals and preferences.

### Limitations

Some limitations of this study should be recognised. The response rate was as expected (~ 20%), but it was not possible to identify any drivers of the preferences. This may be related to the narrow inclusion criteria (RA < 5 years and previous inadequate response to first-line monotherapy). The basis for the selected inclusion criteria was to assess ‘proxy’ preferences of patients ‘eligible for personalizing their treatment’ in patient-doctor communication. By revealing preferences of relatively newly diagnosed patients with RA that had previous experience in changing treatment pathway due to inadequate response. Because common clinical practice in Sweden is to initiate treatment with Methotrexate once the RA diagnosis is established. Patients will need to change treatment pathway if they experience inadequate response to treatment effect or if they experience unbearable side effects. Therefore, the results from this study should be considered to be representative of the targeted patient population, not the general RA population in Sweden. The preference patterns (of the latent class analysis) could not be associated with any of the demographic questions (i.e., age, gender, occupation, education, numeracy, health literacy, RA duration, treatment, side effects, health status, pain, worry and compliance). A possible explanation may be that the sample was too small. Quantitative preference elicitation studies with greater sample sizes may be able to find associations between patient characteristics and preferences. The results may not be generalizable to other countries as the health care systems are different. Aspects of costs and accessibility were not included in this study because treatment of RA is covered by the reimbursement system in Sweden. Further research is needed to develop intervention programmes to support patients in making shared treatment decisions in precision medicine.

In summary, this study revealed that patients have different views on what is most important in RA treatment. This study highlights the importance of understanding the heterogeneity of patient preferences. Understanding how patients weigh treatment goals against one another can inform patient-physician communication in making treatment decisions and can identify patient education needs regarding RA treatment alternatives.

## Conclusions

Patients with RA (< 5 years) and inadequate response to first-line monotherapy have differing treatment preferences. They focus mainly on increasing functional capacity or on the likelihood of getting a severe side effect. Therefore, communication in shared decision-making needs to account for heterogeneity in patient preferences in order to increase patient-centeredness in personalized precision medicine. Future research needs to develop tools to strengthen patients in making shared decisions with health care professionals in order to improve personalized precision medicine.

## Supplementary Information

**Additional file 1. Table 1.** Latent class analysis with covariates tested for potential impact on class membership.

## Data Availability

Data from the current study are available from the corresponding author on reasonable request.
