# Supplementary material for: Physical function and severe side effects matter most to patients with RA (< 5 years): a discrete choice experiment assessing preferences for personalized RA treatment
Source: BMC Rheumatol. 2023 Jul 3;7:17. doi: 10.1186/s41927-023-00341-y (PMC10316588; doi:10.1186/s41927-023-00341-y)
Supplement: Supplementary file 1 — Additional file 1. Table 1. Latent class analysis with covariates tested for potential impact on class membership. [file 41927_2023_341_MOESM1_ESM.pdf]

Physical function and severe side effects matter most to patients with RA (<5 years): A discrete choice experiment assessing preferences for personalized RA treatment

This supplementary file presents the Latent Class Analysis in which demographic and disease-related variables were tested individually for their potential impact on class membership: age, gender, occupation, education, numeracy, health literacy, RA duration, treatment, side effects, health status, pain, worry and compliance (table 1).

Table 1. Latent class analysis with covariates tested for potential impact on class membership.

**Attribute levels**	**Class 1 estimate (SE)**	***P* value**	**CI**	**Class 2 estimate (SE)**	***P* value**	**CI**
**Latent class analysis without covariates**						
**Increase in physical functional capacity**						
25% improvement (ref[a])						
50% improvement	1.0 (0.68)	N/A[b]	-0.334 – 2.328	1.98 (0.48)	<0.01	1.041 – 2.922
75% improvement	2.08 (0.70)	<0.01	0.712 – 4.453	2.95 (0.52)	<0.01	1.931 – 3.972
100% improvement	2.36 (0.81)	<0.01	0.769 – 3.949	3.81 (0.60)	<0.01	2.624 – 4.986
**Increase in psychosocial functional capacity**						
25% improvement (ref)						
50% improvement	0.11 (0.42)	N/A	-0.71 – 0.94	0.68 (0.25)	<0.01	0.19 – 1.16
75% improvement	-0.12 (0.41)	N/A	-0.92 – 0.67	1.12 (0.29)	<0.01	0.56 – 1.68
100% improvement	0.45 (0.44)	N/A	-0.41 – 1.30	1.36 (0.27)	<0.01	0.82 – 1.89
**Frequency of mild side effects**						
Low (ref)						
Medium	-0.13 (0.28	N/A	-0.68 – 0.40	-0.46 (0.16)	<0.01	-0-77 – (-0.14)
High	-0.61 (0.31)	<0.05	-1.21 – (-0.00)	-1.29 (0.21)	<0.01	-1.69 – (-0.88)
**Likelihood of severe side effects**						
Rare: 1 in 1,000 (ref)						
Common: 1 in 100	-0.94 (0.42)	<0.05	-1.76 – (-0.11)	-0.38 (0.23)	N/A	-0.83 – 0.07
Very common: 1 in 10	-4.69 (1.09)	<0.01	-6.82 – (-2.56)	-1.17 (0.31)	<0.01	-1.77 – (-0.07)
**Class probability analysis. Separately calculated for each covariate**						
Age	0.07 (0.14)	0.62	-0.21 – 0.35	-	-	-
Gender	-0.21 (0.39)	0.58	-0.97 – 0.54	-	-	-
Occupation	-0.00 (0.60)	0.97	-0.11 – 0.11	-	-	-
Educational level	0.07 (0.09)	0.46	-0.12 – 0.26	-	-	-
Numeracy	-0.00 (0.00)	0.06	-0.00 – 0.00	-	-	-
Health literacy	-0.00 (0.00)	0.43	-0.00 – 0.00	-	-	-
RA duration	0.03 (0.19)	0.86	-0.35 – 0.42	-	-	-
Treatment (ever prescribed DMARDs)	-0.03 (0.07)	0.63	-0.18 – 0.11	-	-	-
Side effects (ever experienced side effects)	-0.02 (0.07)	0.72	-0.18 – 0.12	-	-	-
Current health status	0.39 (0.37)	0.29	-0.34 – 1.13	-	-	-
Experience of pain	0.43 (0.44)	0.33	-0.44 – 1.30	-	-	-
Experience of worry	0.00 (0.32)	0.98	-0.62 – 0.63	-	-	-
Compliance (How often do you take your treatment based on prescriptions from rheumatologists?)	-0.03 (0.32)	0.91	-0.67 – 0.60	-	-	-
Average class probability	43			57		
[a] Reference category.<br>[b] N/A: not applicable.<br>- Reference estimates						
